# Ddx21 mutant peptide is an effective neoantigen in prophylactic lung cancer vaccines and activates long-term anti-tumor immunity

**DOI:** 10.3389/fimmu.2025.1500417

**Published:** 2025-02-06

**Authors:** Zhe Zhang, Yimeng Xia, Zhihong Wang, Yaxing Sun, Dan Pu, Yijia He, Ruixian Liu, Yanru Zhang, Yan Liu, Junzhi Yu, Shiyang Ning, Baisui Feng, Yaohe Wang, Na Wang

**Affiliations:** ^1^ Department of Gastroenterology, the Second Affiliated Hospital of Zhengzhou University, Zhengzhou, China; ^2^ Department of Microbiology and Immunology, School of Basic Medical Sciences, Zhengzhou University, Zhengzhou, China; ^3^ Centre for Biomarkers and Biotherapeutics, Barts Cancer Institute, Queen Mary University of London, London, United Kingdom

**Keywords:** lung cancer, prophylactic vaccine, neoantigen, Ddx21, central memory T cells

## Abstract

**Introduction:**

Lung cancer is the leading cause of cancer-related death worldwide, and its morbidity and mortality are increasing. Although low-dose CT lung cancer screening has been shown to reduce lung cancer mortality, its adoption rate is limited and the pace of its promotion is slow, highlighting the urgent need for more effective prevention measures. Prophylactic vaccines play a crucial role in cancer prevention. Our previous studies indicated that mice immunized with a prophylactic vaccine based on lung cancer cell lines KPL 160302S, derived from early-stage murine lung cancer tissues, exhibited a significantly extended survival period, with a strong anti-tumor immune response. While the vaccine based on KPL 160424S, derived from advanced-stage murine lung cancer tissues, failed to extend survival time and demonstrated limited capacity to stimulate anti-tumor immunity.

**Methods:**

To investigate the fundamental reason for the difference between KPL 160302S and KPL 160424S vaccines, we employed bioinformatics methods and immune related experiments to explore the effects and mechanisms of the screened neoantigens.

**Results:**

Our findings demonstrated that immunization with the Ddx21 mutant peptide (Ddx21MT), unique to KPL 160302S, could significantly increase the proportion of central memory T cells (TCM) in mice and activate anti-tumor immunity.

**Discussion:**

These results suggest that the Ddx21MT is a highly effective neoantigen that can activate anti-tumor immunity, which can serve as an important component in developing a lung cancer vaccine and is expected to be used in combination with other immunotherapy approaches.

## 1 Introduction

Among all malignant tumors worldwide, lung cancer ranks first in incidence, accounting for 12.4%; as well as first in mortality, accounting for 18.7% (1). One potential factor contributing to the development and progression of cancer is the failure of the immune system to identify and eliminate cancerous cells (2, 3). Immunotherapeutic approaches for cancer, such as checkpoint inhibitors, chimeric antigen receptor T cell (CAR-T) therapy, and cancer vaccines, have demonstrated significant potential in clinical trials (4). Targeted PD-1 or PD-L1 immune checkpoint inhibitors, whether combined with chemotherapy or as a monotherapy, have gained widespread acceptance as first-line treatment of non-small cell lung cancer (NSCLC) (5). However, clinical trials of checkpoint inhibitors have demonstrated limited extension of survival for a minority of patients, with the majority showing no response (6). Furthermore, mutations in cancer therapeutic targets can greatly affect drug sensitivity, and mutation-driven resistance is common in cancer, such as resistance to HER2 and its inhibitors, and resistance to ALK and different generations of ALK inhibitors (7). Chimeric antigen receptor T cell (CAR-T) therapies targeting tumor-specific antigens have certain drawbacks, such as tumor exotoxicity, and have been unable to effectively inhibit malignant cells in some solid tumors due to the presence of various antigens, their expression levels, immunosuppressive environment, and the construction of CARs (8). Hence, it is imperative to investigate more universally applicable immunotherapies.

Vaccines, a common immunization method, can activate the adaptive immune response towards the target antigen, thereby inducing a long-term specific immune response against tumors. Therapeutic vaccines and prophylactic vaccines are available for cancer, but there is a limited availability of prophylactic vaccines for most non-viral malignant tumors. For instance, the majority of lung cancer vaccines primarily function as therapeutic vaccines. There are four main types of non-small cell lung cancer vaccines available, including antigen-targeting vaccines, whole-cell vaccines, carrier-based vaccines, and individual genotype-based vaccines. These vaccines all target advanced lung cancer, and some vaccine trials have shown an immune response after vaccination, usually by targeting an increase in specific cytotoxic T cells. For example, the vaccine targeting Mucin1 and the allogeneic whole tumor cell vaccine Belagenpumatucel-L consisting of four non-small cell lung cancer cell lines transfected with a human transforming growth factor (TGF)-β2 antisense gene expression vector (9). Unfortunately, this did not translate into a significant survival advantage in the Phase III clinical trial. Given the high incidence and mortality rate of lung cancer, it is crucial to prioritize prophylactic measures for this disease. Early detection of lung cancer can significantly reduce mortality rates, indicating that early intervention may offer substantial benefits for high-risk populations (10). Additionally, HLA-LOH (heterozygous deletion) tumor cells often survive during the evolution of lung cancer by evading immune surveillance. Losing the ability to present neoantigens through HLA potentially impacts the effectiveness of therapeutic vaccines for advanced lung cancer (11). Vaccines derived from novel antigens generated in the early stages of tumor progression may yield superior outcomes.

One of the defining features of cancer is the accumulation of genetic mutations, leading to the emergence of cancer-specific neoantigens. Neoantigens can also arise from viral infections, alternative splicing, and genetic rearrangement (12). Epigenetic changes in cancer include DNA methylation, histone modification, and chromatin remodeling (13). Epigenetic therapy can remove epigenetic inhibition on the transposon sequence, reactivate the promoter activity of the transposon, and thus produce TE-gene chimeric transcripts (TETs), which may be translated into chimeric proteins. It also has the potential to provide neoantigens (14). These neoantigens are recognized as foreign substances by autologous T cells (15) and are considered as the target of tumor immunotherapy due to their high tumor specificity and immunogenicity (16). Therefore, the research on neoantigens in prophylactic lung cancer vaccines plays a key role in the development of vaccines.

The most common mutations in human NSCLC include activation of *Kras* (10-30%) and functional loss of *p53* (50-70%) (17, 18). We used Ad-Cre administered intranasally to induce the activation of the *Kras* and *p53* mutant genes in *Kras^LSL-G12D/+^; p53^LSL-R172H/+^* mice (129 mice with *KRAS^LSL-G12D/+^; p53^LSL-R172H/+^*) to establish a lung cancer model that maximally simulates the process of lung cancer development triggered by *Kras* and *p53* mutations in humans. Our previous research has demonstrated that the lung cancer cell lines KPL 160302S, obtained from *Kras^LSL-G12D/+^*/*p53^LSL-R172H/+^* mice with early-stage lung cancer, and KPL 160424S, obtained from *Kras^LSL-G12D/+^*/*p53^LSL-R172H/+^* mice with later-stage lung cancer, display distinct efficacy as prophylactic whole tumor cell vaccines for immunizing lung cancer mice. A vaccine based on the KPL 160302S cell line demonstrates a significant capacity to prolong the survival period of lung cancer mice and elicit a robust anti-tumor immune response. In contrast, the vaccine based on the KPL 160424S cell line does not confer protective effects in this model (19). Identifying the key neoantigens responsible for the disparities in anti-tumor immune responses between these two cell lines is crucial for enhancing the effectiveness of prophylactic vaccines against lung cancer. After conducting whole-exome sequencing and RNA sequencing on KPL 160302S and KPL 160424S, followed by bioinformatics analysis, we identified 12 candidate neoantigens specific to KPL 160302S. IFNγ release assay *in vitro* showed that Ddx21MT (SNFPIFCDL) and Zfp760MT (KCFRSYSSL) peptides could induce significant production of IFNγ by splenocytes from mice immunized with the KPL 160302S based vaccine, indicating that these 2 neoantigens are immunogenic. Subsequent research demonstrated that Ddx21MT peptide immunization could significantly up-regulate TCM in mice and activate anti-tumor immunity.

## 2 Materials and methods

All animal procedures were approved by the Ethics Committee of Henan Key Laboratory of Hepatology Pharmacology Animal (Zhengzhou, China). Mice were housed in groups in accordance with the regulations for welfare and ethics of the Ethics Committee of Henan Key Laboratory of Hepatology Pharmacology with 12h dark-light cycles and free access to food and water.

### 2.1 Procedures for the preparation of KPL 160302S and KPL 160424S lung cancer cell vaccines and immunization protocol for *Kras^LSL-G12D/+^*/*p53^LSL-R172H/+^* mice

KPL 160302S and KPL 160424S were infected with Ad5 (MOI: 50 PFU/cell) for 4 hours and then incubated with mitomycin C for 2.5 hours as the primary immunization agent. KPL 160302S and KPL 160424S lung cancer cell vaccines were inoculated with 100μL (5×10^6^cells) per mouse in the right dorsal subcutaneous area of *Kras^LSL-G12D/+^*/*p53^LSL-R172H/+^* male mice 2 weeks and 4 days after Ad-Cre administration as primary immunization. 4 weeks later, KPL 160302S and KPL 160424S were infected with VVLl5-RFP (MOI: l PFU/cell) for 2 hours and incubated with mitomycin C for 2.5 hours as a booster vaccine to inoculated, on the same subcutaneous side for booster immunity, 100μL (5×10^6^cells) per mouse.

### 2.2 Flow cytometry was used to detect immune cell subsets in spleens of *Kras^LSL-G12D/+^*/*p53^LSL-R172H/+^* mice immunized with lung cancer cell vaccine

2 weeks and 4 days after Ad-Cre administration, KP male mice were given primary immunization and booster immunization according to the method described in 2.1. After 4 weeks of booster immunization, the splenic cells were stained with flow antibody CD3e(FITC), CD4(APC), CD8a(PE), CD44(eFluor 450), CD62L(PerCP-Cyanine5.5) for flow cytometry test.

### 2.3 Whole exome sequencing of KPL 160302S/KPL 160424S cells

Genomic DNA of KPL 160302S cell line, KPL 160424S cell line, and peripheral blood of KP mice after Ad-Cre administration were extracted. Whole-exome sequencing was performed in Shenzhen BGI Co. LTD. The data of whole-exome sequencing has been uploaded to SRA, the series entry number is PRGNA1124348.

### 2.4 Transcriptome sequencing of KPL 160302S and KPL 160424S cells (RNA-seq)

After the KPL 160302S and KPL 160424S cells were confirmed to be free of mycoplasma contamination, they were treated with Trizol, and transcriptome sequencing was performed at BGI Co. LTD in Shenzhen. The data of transcriptome sequencing has been uploaded to GEO, the series entry number is GSE151813.

### 2.5 KPL 160302S candidate neoantigen prediction

Sequence comparison and processing are described in the literature (20). To obtain somatic mutations in KPL 160302S and KPL 160424S, we introduced the bcbio-nextgen (version 1.1.1a0) tumor mutation call pipeline, configured with a BWA and two mutation callers (VarScan and Verdict). The final mutation set was optimized using the bcbio integration algorithm of numpass 1. The mutant 8-11 amino acid sequences were sorted by NetMHCpan for their affinity to MHC-I H-2D^b^ and H-2K^b^ molecules. Somatic mutations in KPL 160302S/KPL 160424S filtered out mutations in the peripheral blood genome induced by Ad-Cre nasal drops in a *Kras^LSL-G12D/+^*/*p53^LSL-R172H/+^* mice to exclude potentially rare germline variants. Because high affinity is beneficial to the efficacy of T cells, mutant peptide segments with semi-inhibitory concentration (IC50) <100nM are selected as candidate neoantigens. The final candidate neoantigens were screened by depth (coverage and VAF) and expression (FPKM). Normal Coverage≥5× (up to 20×), Normal VAF ≤ 2%, Tumor Coverage (DNA and RNA) ≥10×, Tumor VAF (DNA and RNA)≥40%, FPKM>1.Finally, 12 neoantigens presented in KPL 160302S but not in KPL 160424S were screened. Since deletion and insertion of large fragments may also produce neoantigens, we do not restrict variant types, so there are individual antigens that do not have corresponding wild-type epitope sequences.

### 2.6 Candidate neoantigen peptides induced IFNγ secretion in 129 mice spleens cells *in vitro*

6-week-old 129 wild-type mice were given KPL 160302S/KPL 160424S/PBS subcutaneously on the right side of the back as primary immunization, and KPL 160302S/KPL 160424S/PBS as booster immunization 2 weeks later. The two immunization schedules are the same as described in the above 2.1 part.1 week after the booster immunization, the spleens were crushed, red blood cells were lysed, and the spleen cells suspension was prepared by filtration through a 70μM filter. 5×10^5^cells/100μL/well were cultured in 96-well plates and incubated with 10μg-peptides/100μL/well. The Background well contained 5× 10^5^ spleen cells in 200 μL T cells medium. These plates were cultured at 37°C for 3 days in a 5% CO_2_ incubator. IFNγ secretion in supernatant was measured by ELISA.

### 2.7 129 mice were immunized with neoantigens

Neoantigen peptide 100μg/50μL and adjuvant Poly-IC 50μg/50μL were mixed to prepare a neoantigen based vaccine. 6-week-old 129 wild-type mice were inoculated subcutaneously on the right side of the back as primary immunization. Two weeks after the primary immunization, the same dose of neoantigen vaccine was inoculated again as the booster immunization at the same location.

### 2.8 Flow cytometry was used to detect 129 mice immune cell subsets after neoantigen immunization

One week after the booster immunization, spleens of mice were harvested to prepare single-cell suspensions. The single-cell suspensions were stained with CD45(PerCP), CD3(PECy7), CD8(FITC), CD4(BV510), CD62L(PE), CD44(APC), CD127(BV711), CD25(BV421). After staining at 4°C, lymphocyte subpopulations were analyzed with BDFACSAria™III cytometry.

### 2.9 *In vitro* induction of IFNγ secretion in splenocytes after immunization with neoantigen vaccine

6-week-old 129 wild-type mice were immunized according to the procedure described in the above 2.7 part. The spleens were collected one week after the booster immunization, and IFNγ secretion in supernatant was measured with the same method in the above 2.6 part.

### 2.10 The prevention of subcutaneous tumor by neoantigen vaccine

6-week-old 129 wild-type mice were immunized according to the procedure described in the above 2.7 part. Each mouse was rechallenge with 1×10^5^ KPL 160302S/KPL 160424S on day 22.

### 2.11 Transcriptome sequencing (RNA-seq) of CD4^+^ T and CD8^+^ T cells in spleens of 129 mice immunized with Ddx21MT neoantigen

6-week-old 129 mice were immunized according to the procedure described in the above 2.7 part. One week after the booster immunization, spleens were collected to prepare single-cell suspensions. CD4^+^ T cells and CD8^+^ T cells were sorted by magnetic beads, and transcriptome sequencing was performed by Shanghai-Weihuan Company. The data of transcriptome sequencing has been uploaded to GEO, the series entry number is GSE270091.

## 3 Results

### 3.1 KPL 160302S tumor cell vaccine can induce an increase of TCM in spleen

**Figure 1:**
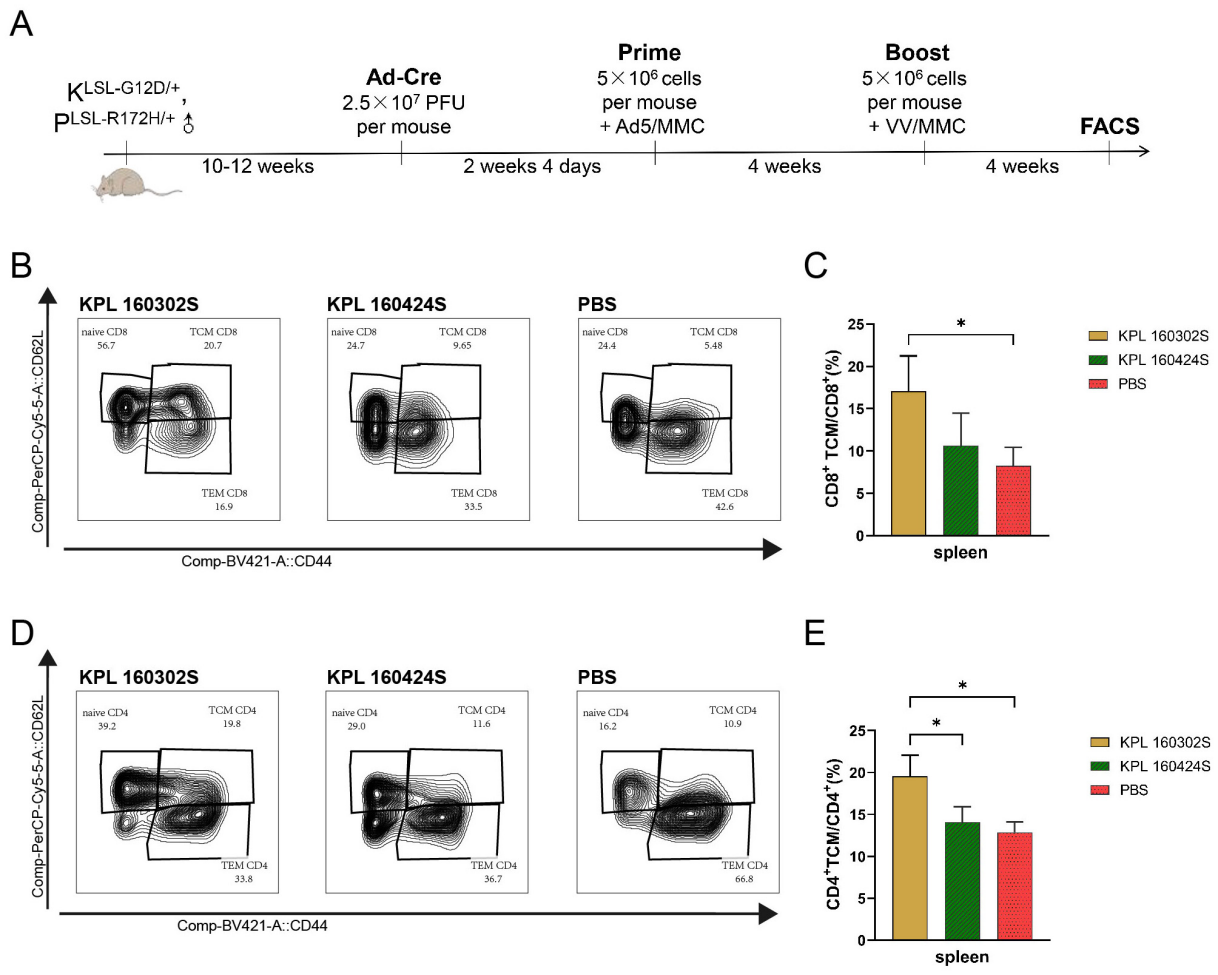
Immune cell subsets in spleens of *Kras^LSL-G12D/+^*/*p53^LSL-R172H/+^* mice immunized with KPL160302S/KPL 160424S tumor cell vaccine. **(A)** KPL 160302S/KPL 160424S tumor cell vaccine immunization scheme for mice. **(B, D)** Representative FACS profiles with CD8^+^ TCM**(B)**/CD4^+^ TCM**(D)** populations as a percentage of CD8^+^ T/CD4^+^ cells in splenocytes of KPL 160302S/KPL 160424S/PBS. **(C, E)** Analysis of TCM CD8^+^ T **(C)**, TCM CD4^+^ T **(E)** cells in spleens after 4 weeks of boosted immunization with 160302S/KPL 160424S tumor cell vaccine (One-way ANOVA and multiple comparisons between groups, n=3, *p< 0.05).

Lung cancer would occur spontaneously in *Kras^LSL-G12D/+^*/*p53^LSL-R172H/+^* mice infected with Ad-Cre via intranasally delivery. At 8 and 16 weeks after nasal instillation, lung tissues were taken out for primary culture. Then, the lung cancer cell lines KPL 160302S from early-stage lung cancer and KPL 160424S from advanced-stage lung cancer were obtained, respectively. We have found in our previous research that immunizing *Kras^LSL-G12D/+^*/*p53^LSL-R172H/+^* mice with KPL 160302S based vaccine could increase the median survival time of mice by 21%, but KPL 160424S based vaccine had no protection in mice (19). In addition, the infiltration of CD8^+^ T and CD4^+^ T cells in lungs was increased to a greater extent and for a longer period in KPL 160302S vaccine group compared to KPL 160424S vaccine group (19). To further investigate the difference in the immune protection mechanisms of tumor cell vaccines based on KPL 160302S and KPL 160424S, we examined the alterations of immune cell subsets in spleens of mice after vaccination. 4 weeks after the booster immunization, the spleens of *Kras^LSL-G12D/+^*/*p53^LSL-R172H/+^* mice were collected and CD4^+^ T cells and CD8^+^ T cells were detected by flow cytometry (**Figure 1A**). The results (**Figures 1B–E**) showed that only the KPL 160302S vaccine significantly increased the number of TCM in CD4^+^ T cells and CD8^+^ T cells of the spleens compared with the PBS group at 4 weeks after booster immunization.

### 3.2 Identification and validation of neoantigens of KPL 160302S

To investigate the differences in immune protection of KPL 160302S based vaccines and KPL 160424S based vaccines for *Kras^LSL-G12D/+^/p53^LSL-R172/+^* mice lung cancer models, we conducted whole-exome sequencing and transcriptome sequencing (RNA-seq) on these two cell lines.

**Figure 2:**
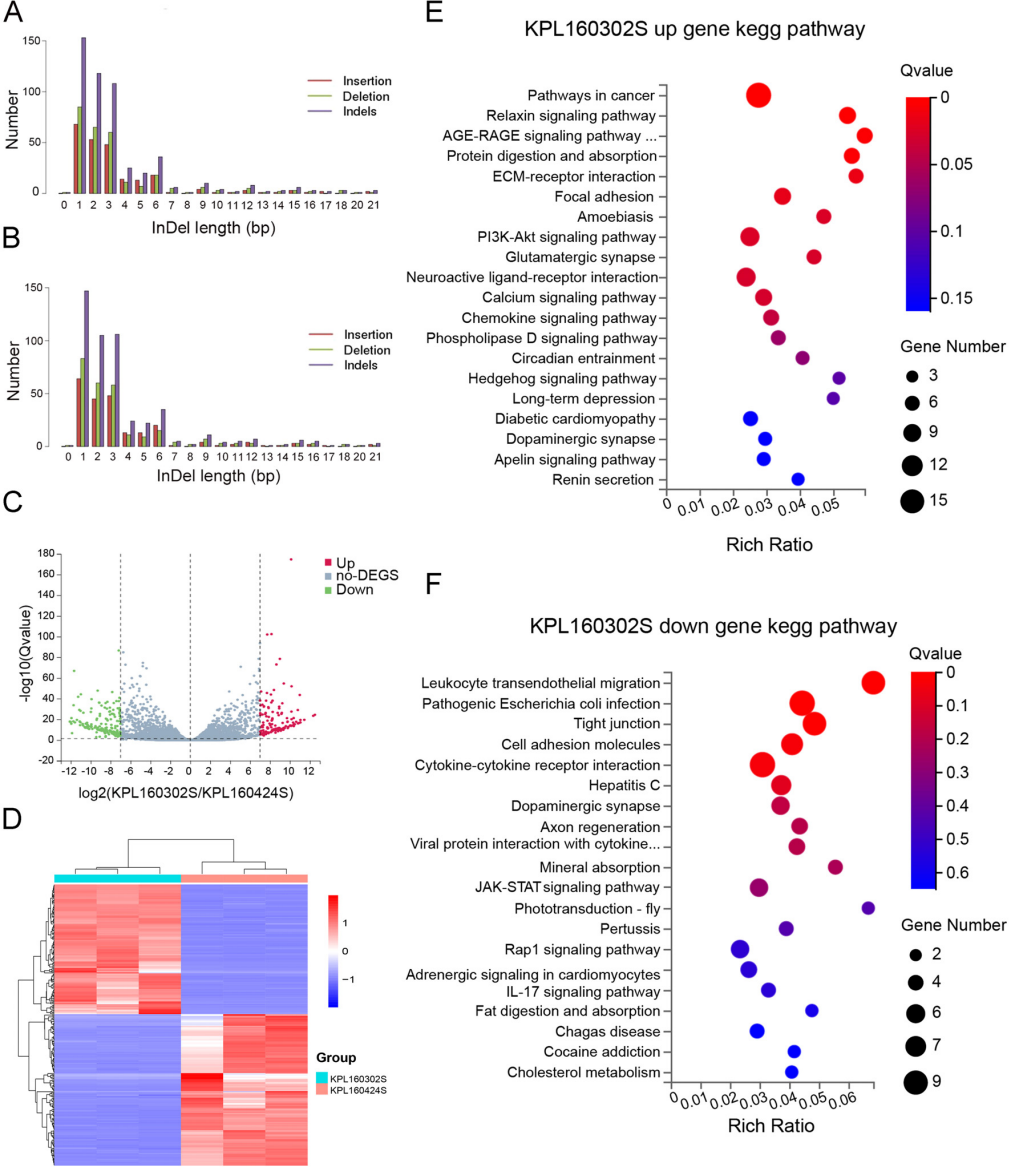
Analysis of whole-exome and transcriptome sequencing for KPL 160302S and KPL 160424S. **(A)** Somatic insertion or deletion mutations in exons of KPL 160302S. **(B)** Somatic insertion or deletion mutations in exons of KPL 160424S. **(C)** Volcanic map of differential genes between KPL 160302S and KPL 160424S cell lines (|log2FC|=7). **(D)** Heat maps of differential genes of the two cell lines (|log2FC|=7, Padj<0.05, the number of differential genes as 310, using Log_2_^(TPM+1)^ as the standard). **(E, F)** Kegg pathway enrichment bubble map of up-regulated and down-regulated gene in KPL 160302S, compared with KPL 160424S.

Whole-exome sequencing revealed insertion and deletion variants in both cell lines (**Figures 2A, B**). The transcriptome sequencing of the two cell lines was performed, and the differentially expressed genes of the two cell lines were analyzed with |log2FC|≥7, padj ≤ 0.05. Genes that are underexpressed are screened out(Average read counts<10). In contrast to the KPL 160424S cell line, there were 313 differential genes including 143 up-regulated genes and 170 down-regulated genes in KPL 160302S (**Figure 2C**). Three low-expressed genes are screened out(Average read counts<10). The cluster heat map analysis of the 310 genes showed that the differential genes had good homogeneity within the group, and the expression levels were significantly different between the two cell lines (**Figure 2D**). Kegg pathway enrichment of differential genes showed that up-regulated genes of the KPL 160302S cell line were mainly enriched in pathway in cancer, AGE-RAGE signaling pathway, ECM-receptor interaction, chemokine signaling pathway, hedgehog signaling pathway and other signaling pathways. KPL 160302S down-regulated genes are mainly enriched in leukocyte transendothelial migration, tight junction, cell adhesion molecules, cytokine-cytokine receptor interaction and other pathways (**Figures 2E, F**).

**Figure 3:**
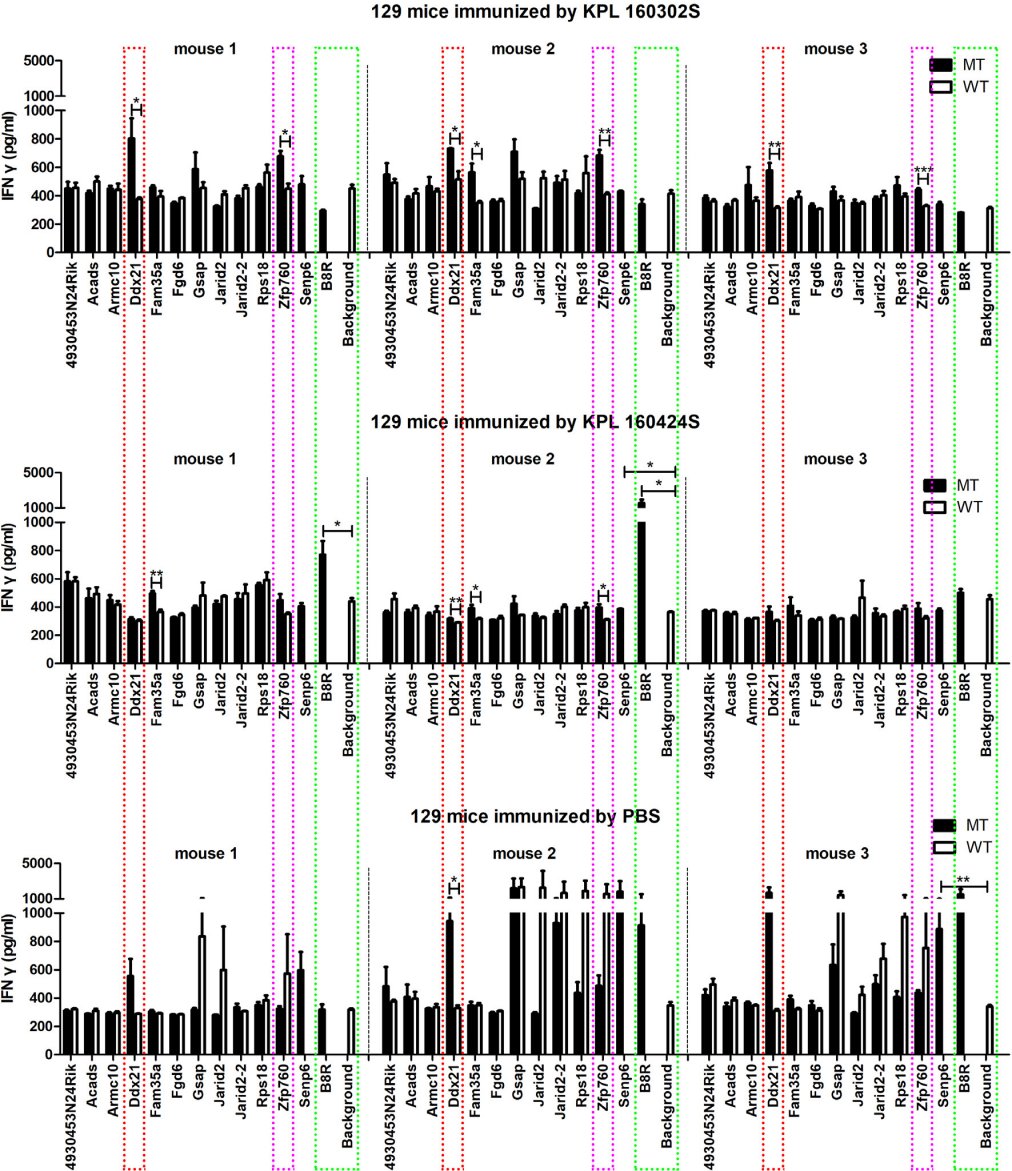
Secretion of IFNγ *in vitro* to neoantigens by spleen cells of 129 mice after immunization of KPL 160302S/KPL 160424S/PBS. MT represents the screened neoantigen peptide segment and WT represents the corresponding wild-type peptide segment(Unpaired T-test, ***p < 0.001, **p < 0.01, *p < 0.05).

RNA-seq showed no difference in MHC-I gene expression between the two cell lines (**Supplementary Figure S1**), suggesting that the difference in immunogenicity between the two cell lines was due to the presence of distinct tumor neoantigens. Therefore, bioinformatics was used to analyze the whole-exome sequencing and transcriptome sequencing of these two cell lines to identify the neoantigens that are specific to both cell lines. Somatic mutations in KPL 160302S/KPL 160424S filter out genomic mutations in the peripheral blood of a *Kras^LSL-G12D/+^*/*p53^LSL-R172H/+^* mouse after Ad-Cre administration to exclude potentially rare germline variation. Due to high affinity is beneficial for the efficacy of T cells (21–23), mutated peptides with half inhibitory concentration (IC50) ≤100 nM were elected as the candidate neoantigens (24). Mutations that also existed in KPL 160424S cell line were removed, and neoantigens produced by mutations only in KPL 160302S cell line were retained. And 12 candidate neoantigens that might be associated with different immune effects caused by the two cell lines were selected (**Table 1**). Since the insertion and deletion of large fragments can also generate potential neoantigens (20), we did not restrict the type of variation. Therefore, there are some neoantigens that do not have corresponding wild-type epitope sequences.To assess the immunogenicity of potential tumor neoantigens, we isolated splenocytes from 129 wild-type mice that had been immunized with KPL 160302S/KPL 160424S/PBS and subsequently stimulated them *in vitro* with candidate neoantigens. The production of IFNγ was then measured. After being vaccinated with KPL 160302S, all three mice exhibited a significant increase in IFNγ secretion when exposed to Ddx21MT and Zfp760MT. Among the three mice immunized with KPL 160424S, only one showed a higher IFNγ response to both Ddx21MT and Zfp760MT, while among the three mice immunized with PBS, only one showed a higher IFNγ response to the Ddx21MT (**Figure 3**). We also observed that the peptide B8R (TSYKFESV) from the vaccinia virus did not significantly enhance IFNγ secretion in all the three immunized mice with KPL 160302S, but it did significantly increase IFNγ secretion in two out of the three mice immunized with KPL 160424S (**Figure 3**). This also indicates that the immunity induces by KPL 160302S vaccine was more targeted towards tumors rather than the adjuvant vaccinia virus. Based on the IFNγ release assay results, we postulate that the prophylactic vaccine derived from the KPL 160302S cell line may confer protection against lung cancer in *Kras^LSL-G12D/+^*/*p53^LSL-R172H/+^* mice, potentially attributed to the generation of immune memory cells elicited by two novel antigens, Ddx21MT and Zfp760MT.

**Table 1:** Candidate neoantigens in KPL 160302S.

Chr	Start	Stop	Reference	Variant	Variant Type	Mutation	Gene Name	MT Epitope Seq	Gene Expression	HLA Allele	Best MT Score
16	64770805	64770806	C	T	missense	D20N	4930453N24Rik	LGLLNNDEI	1552	H-2-Db	43.1
5	115113142	115113143	T	C	missense	D94G	Acads	LGYLAYSIAL	1107	H-2-Kb	53.1
5	21661393	21661394	G	A	missense	G233S	Armc10	SQVANEILL	1811	H-2-Db	68.8
10	62595062	62595062	G	GAAATGGGATCGGAAAGCTCCCTCTTTCTGCTCCACAGGTATTTCCTATCAAAGCAAAAGATCACAGA	FS	S266FCDLLL**EIPVEQKEGAFRSHFX	Ddx21	SNFPIFCDL	11593	H-2-Kb	43.7
14	34268822	34268823	A	G	missense	F42S	Fam35a	LSYKQHSL	653	H-2-Kb	58.4
10	94044326	94044327	T	A	missense	C348S	Fgd6	YSLKNNKVSVL	2882	H-2-Db	71.6
5	21246975	21246976	G	A	missense	D339N	Gsap	SQVTNGIAFL	779	H-2-Db	86.6
13	44823426	44823427	A	T	missense	K31N	Jarid2	RNVLYLSL	884	H-2-Kb	28.6
13	44823426	44823427	A	T	missense	K31N	Jarid2	RVVRNVLYL	884	H-2-Db	60.8
17	33952576	33952577	T	G	missense	R39S	Rps18	RSYAHVVL	44030	H-2-Kb	35.3
9	80136749	80136750	GA	G	FS	K888X	Senp6	ASGMNASVL	5576	H-2-Db	75.9
17	21722419	21722420	G	A	missense	C192Y	Zfp760	KCFRSYSSL	1440	H-2-Kb	89.4

* means termination codon.

### 3.3 Ddx21MT serves as an effective neoantigen in the vaccine, facilitating the generation of memory immune cells

**Figure 4:**
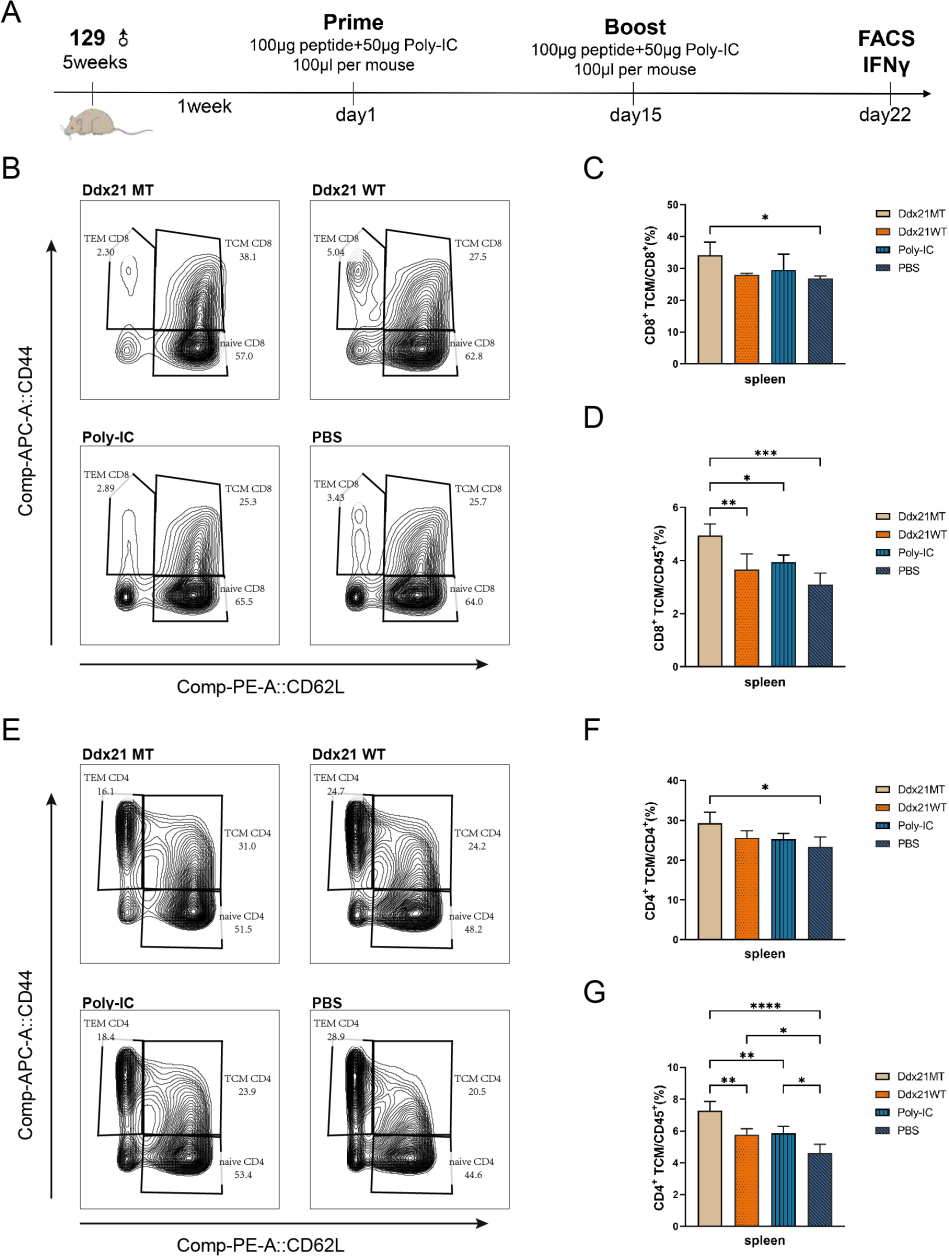
Analysis of T cell subpopulations in spleens of 129 mice one week after immunization with two neoantigens and corresponding wild-type peptides. **(A)** Protocol for immunizing 129 mice with neoantigens or corresponding wild-type peptides. **(B, E)** Representative FACS profiles with CD8^+^ TCM **(B)**/CD4^+^ TCM **(E)** populations as a percentage of CD8^+^/CD4^+^ cells in splenocytes of 129 mice one week after immunization with Ddx21 neoantigens and corresponding wild-type peptides. **(C)** Proportion of CD8^+^ TCM cells among CD8^+^ cell population in spleens. **(D)** Proportion of CD8^+^ TCM cells among CD45^+^ cell population in spleens. **(F)** Proportion of CD4^+^ TCM cells among CD4^+^ cell population in spleens. **(G)** Proportion of CD4^+^ TCM cells among CD45^+^ cell population in spleens. (One-way ANOVA and multiple comparisons between groups, n=4, ****p < 0.0001, ***p < 0.001, **p < 0.01, *p < 0.05).

After one week of booster immunization with the Ddx21MT peptide co-administered with Poly-IC adjuvant in 129 wild-type mice (**Figure 4A**), there is a significant up-regulation of TCM populations of CD8^+^ T and CD4^+^ T cells in the spleens compared to the PBS group (**Figures 4B, C, E, F**, **Supplementary Figure S2**), consistent with the immune effect induced by KPL 160302S vaccination (**Figure 1**). In addition, the proportions of CD8^+^ TCM and CD4^+^ TCM in CD45^+^ cells, a larger gate, of Ddx21MT group are significantly higher than Ddx21WT, Poly-IC and PBS group (**Figures 4D, G**). We also demonstrated a significant effect of the vaccine on the formation of TCM in the spleens in B6 mice with a consistent MHC haplotype (**Supplementary Figure S3**). However, immunization of mice with the Zfp760MT peptide combined with Poly-IC adjuvant did not result in a significant up-regulation of TCM in both CD8^+^ T and CD4^+^ T cell populations(not shown). Based on these findings, we postulate that the Ddx21MT peptide may represent a novel and effective antigen capable of facilitating the generation of memory T cells in mice.

**Figure 5:**
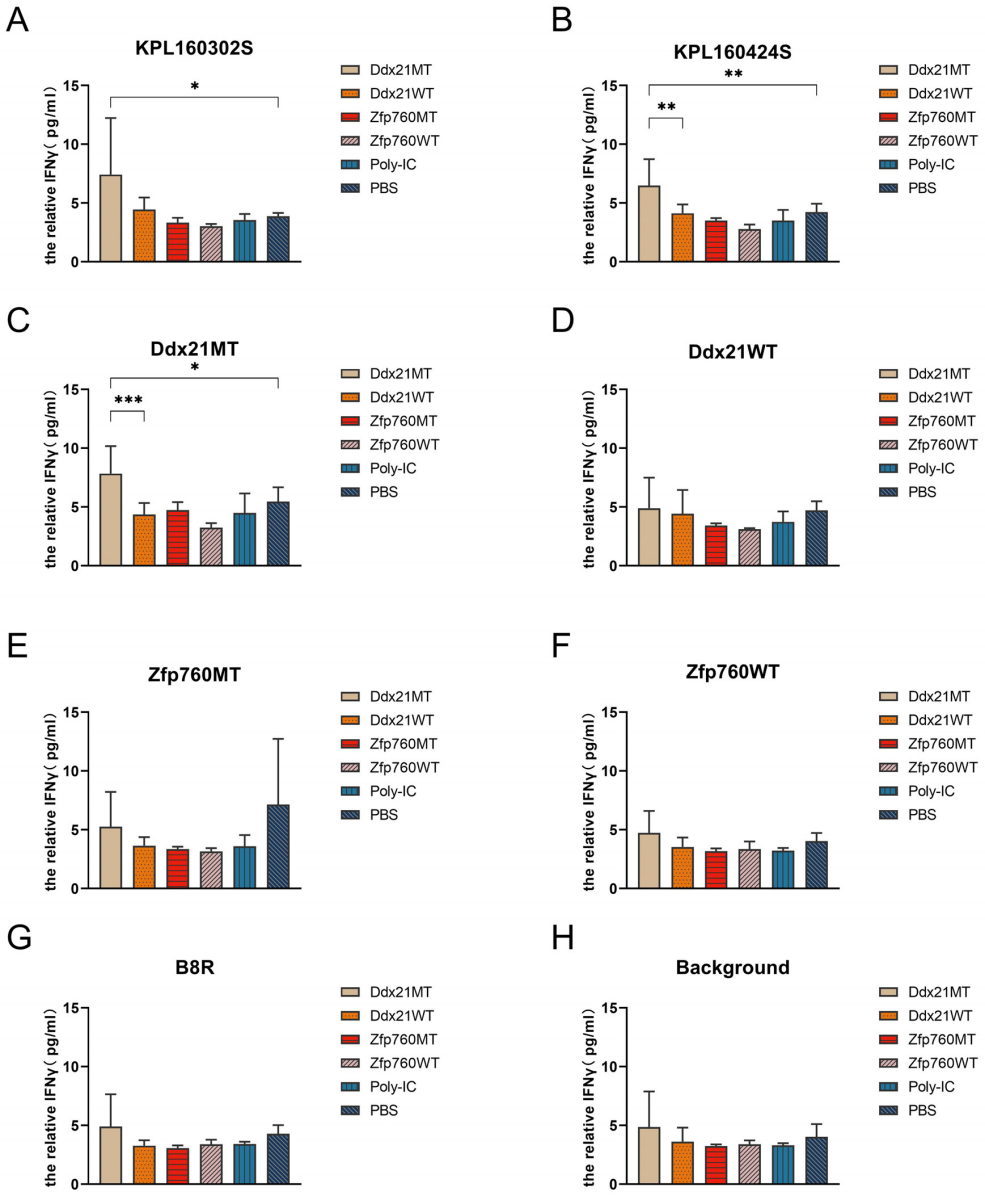
IFNγ secretion of splenocytes from 129 mice immunized with two neoantigens. **(A)** IFNγ secretion of splenocytes stimulated by KPL 160302S cells. **(B)** IFNγ secretion of splenocytes stimulated by KPL 160424S cells. **(C)** IFNγ secretion of splenocytes stimulated by Ddx21MT peptide. **(D)** IFNγ secretion of splenocytes stimulated by Ddx21WT peptide. **(E)** IFNγ secretion of splenocytes stimulated by Zfp760MT peptide. **(F)** IFNγ secretion of splenocytes stimulated by Zfp760WT peptide. **(G)** IFNγ secretion of splenocytes stimulated by B8R peptide. **(H)** blank control (One-way ANOVA and multiple comparisons between groups,n=4, ***p < 0.001, **p < 0.01, *p < 0.05).

To further investigate the impact of the neoantigen on T cell function, we isolated splenocytes from mice that had been immunized with the neoantigen and co-cultured them *in vitro* with KPL 160302S and KPL 160424S cell lines, as well as Ddx21MT and Zfp760MT respectively. Subsequently, we measured the secretion of IFNγ. The results indicated that splenocytes from mice immunized with Ddx21MT produce higher levels of IFNγ in response to stimulation by KPL 160302S, KPL 160424S, and the Ddx21MT peptide (**Figure 5**). However, the splenocytes of mice immunized with Zfp760MT could not be stimulated to produce higher level of IFNγ secretion by lung cancer cells or candidate neoantigens. The results further confirmed that the Ddx21MT peptide could stimulate the generation of memory T cells in mice, making it a key neoantigen in the KPL 160302S based tumor cell vaccine.

### 3.4 Ddx21MT vaccine could delay the growth of KPL 160302S subcutaneous tumor

**Figure 6:**
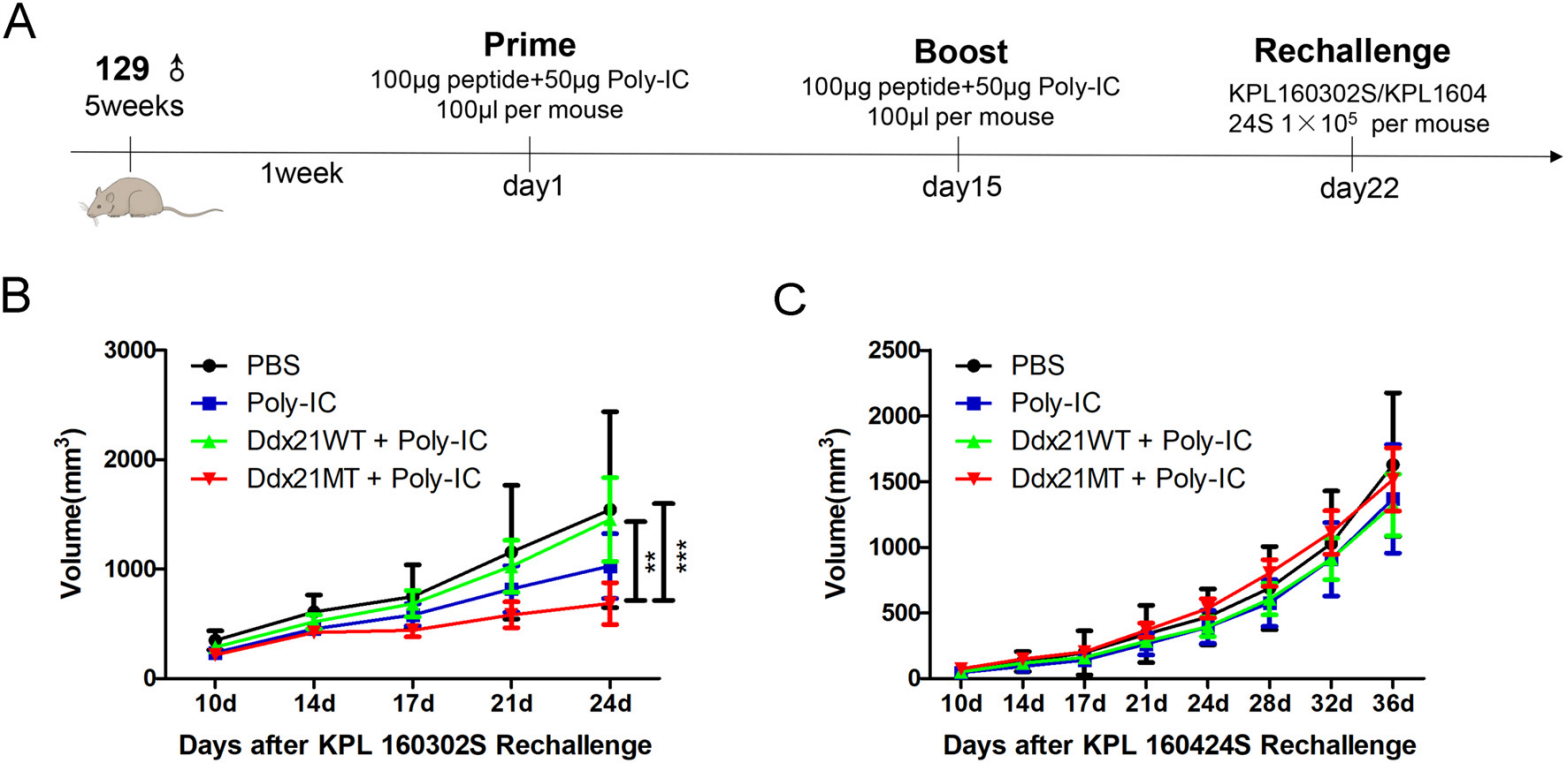
Experimental effect of neoantigen vaccine on the prevention of subcutaneous tumors in 129 mice. **(A)** Neoantigen or corresponding wild-type peptide vaccine for subcutaneous tumor prevention in 129 mice. **(B)** The volume change of subcutaneous tumor formed by KPL 160302S after immunization with neoantigen and its wild peptide vaccine (n=6-8). **(C)** The volume change of subcutaneous tumor formed by KPL 160424S after immunization with neoantigen and its wild peptide vaccine (n=5-7). (2-way ANOVA with Bonferroni *post-hoc* testing, ***p < 0.001, **p < 0.01).

129 wild-type mice were inoculated with KPL 160302S and KPL 160424S subcutaneous tumors after 1 week of enhanced immunization with Ddx21MT peptide and Poly-IC adjuvant (**Figure 6A**). After immunization with Ddx21MT+Poly-IC vaccine, the subcutaneous tumor volume of KPL 160302S was significantly reduced, while immunization with Ddx21WT+Poly-IC and Poly-IC had no such effect (**Figure 6B**). However, Ddx21MT +Poly-IC vaccine did not reduce tumor volume of KPL 160424S (**Figure 6C**). *In vivo* experiments confirmed that Ddx21MT vaccine had a significant effect on the prevention of KPL 160302S with the same mutation.

### 3.5 Transcriptome sequencing (RNA-seq) further elucidates the molecular mechanism of Ddx21MT promoting the formation of immune memory cells in mice

**Figure 7:**
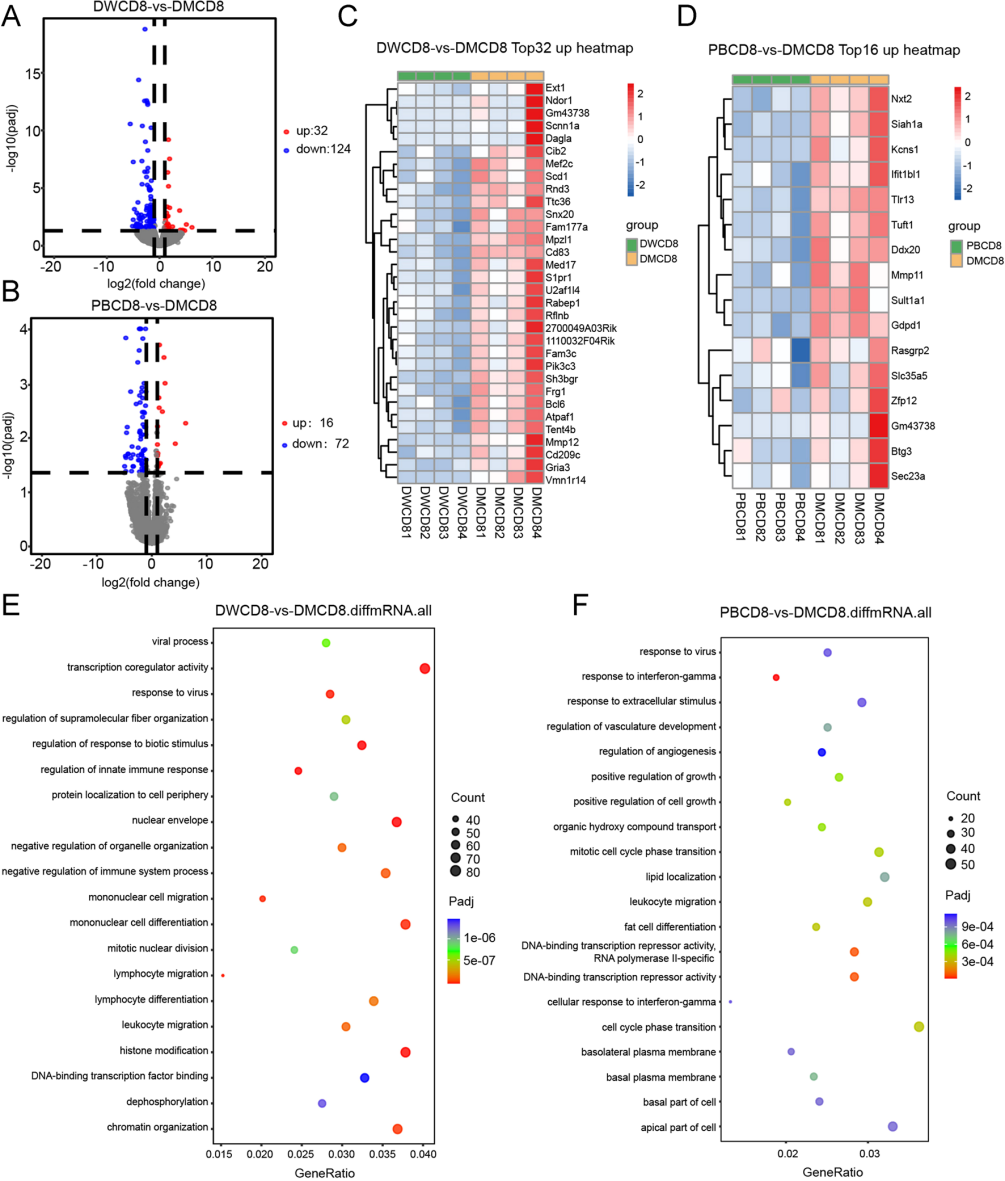
mRNA sequencing of CD8^+^ T cells in splenocytes of 129 mice one week after immunized with Ddx21MT (|log2FC|≥1, padj < 0.05). **(A)** Volcano map of CD8^+^ T cells expression differential genes of Ddx21MT group relative to Ddx21WT group. **(B)** Volcano map of CD8^+^ T cells expression differential genes of Ddx21MT group relative to PBS group. **(C)** Heat map of CD8^+^ T cells expressed 32 up-regulated genes of Ddx21MT group relative to Ddx21WT group. **(D)** Heat map of CD8^+^ T cells expressed 16 up-regulated genes of Ddx21MT group relative to PBS group. **(E)** GO enrichment pathway of CD8^+^ T cells expression differential genes of Ddx21MT group compared to Ddx21WT group. **(F)** GO enrichment pathway of CD8^+^ T cells expression differential genes of Ddx21MT group compared to PBS group.

**Figure 8:**
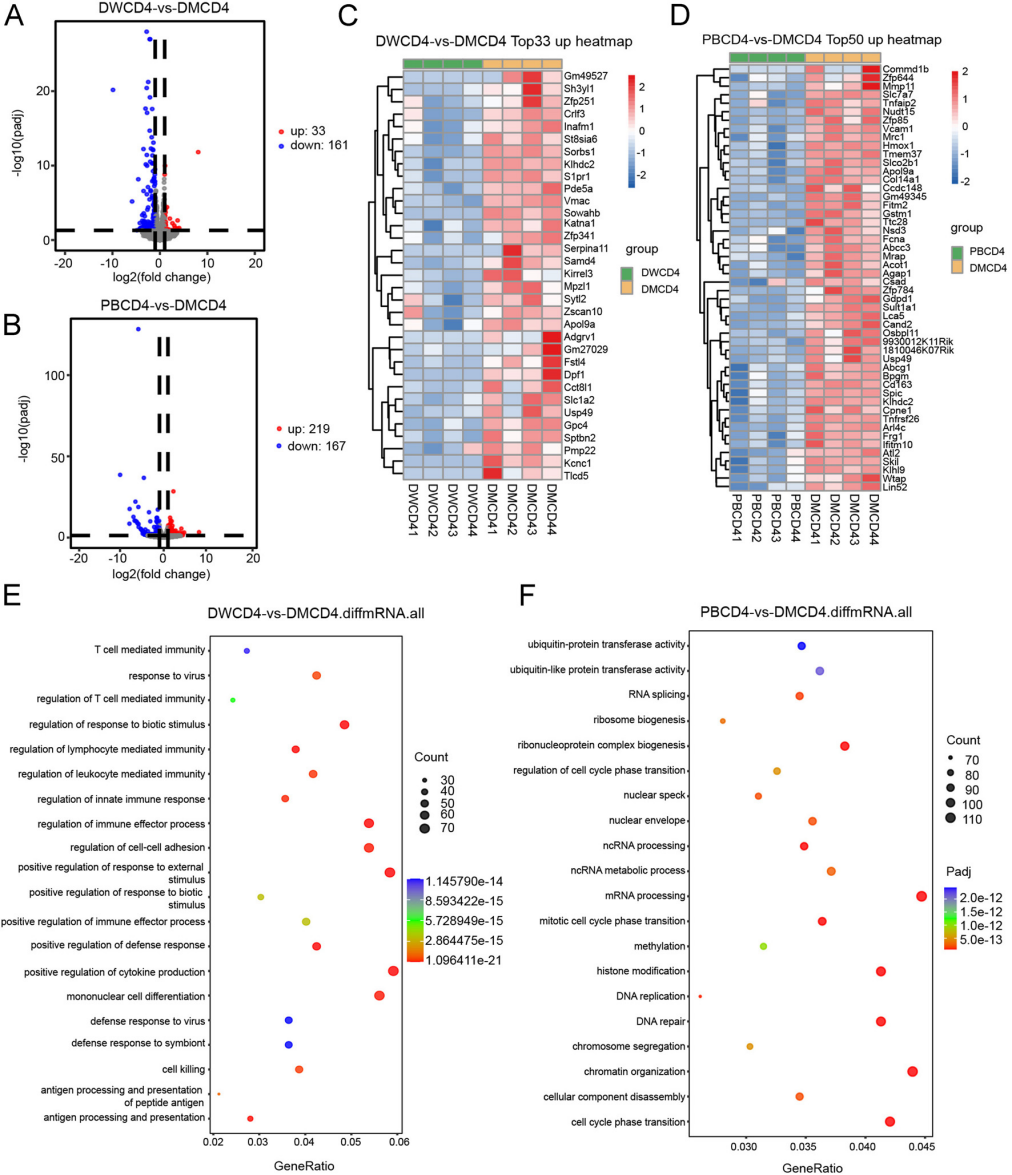
mRNA sequencing of CD4^+^ T cells in splenocytes of 129 mice one week after immunized with Ddx21MT (|log2FC|≥1, padj < 0.05). **(A)** Volcano map of CD4^+^ T cells expression differential genes of Ddx21MT group relative to Ddx21WT group. **(B)** Volcano map of CD4^+^ T cells expression differential genes of Ddx21MT group relative to PBS group. **(C)** Heat map of CD4^+^ T cells expressed 33 upregulated genes of Ddx21MT group relative to Ddx21WT group. **(D)** Heat map of CD4^+^ T cells expressed 50 upregulated genes of Ddx21MT group relative to PBS group. **(E)** GO enrichment pathway of CD4^+^ T cells expression differential genes of Ddx21MT group compared to Ddx21WT group. **(F)** GO enrichment pathway of CD4^+^ T cells expression differential genes of Ddx21MT group compared to PBS group.

To further study the mechanism of Ddx21MT peptide promoting immune response in mice, CD8^+^ T and CD4^+^ T cells in the spleens of 129 mice after Ddx21MT peptide immunization were selected for transcriptome sequencing. According to the volcano map and heat map (**Figures 7A–D**, **8A–D**), both CD8^+^ T cells and CD4^+^ T cells in the spleens of the mice immunized with Ddx21MT, showed significant differences in gene expression compared to CD8^+^ T and CD4^+^ T cells from the mice immunized with Ddx21WT or PBS. We compared the up-regulated genes of CD8^+^ T and CD4^+^ T cells in the Ddx21MT group with the other two control groups and found that *S1pr1, Mpzl1, Mmp11, Sult1a1, Gdpd1*, and other genes were up-regulated both in CD8^+^ T and CD4^+^ T cells.

In addition, GO enrichment analysis of these differential genes in CD8^+^ T cells of Ddx21MT group (**Figures 7E, F**) were enriched in monocyte differentiation and migration, lymphocyte differentiation and migration, leukocyte migration, and histone modification, as well as in regulation of innate immune response, positive regulation of cell growth, cellular response to IFNγ, and other pathways. Compared with Ddx21WT group and PBS group, CD4^+^ T in Ddx21MT group (**Figures 8E, F**) was differentially expressed in T cell mediated immunity, regulation of lymphocyte and leukocyte mediated immunity, antigen processing and presentation, positive regulation of biological response and defense response, positive regulation of cytokine production, cell cycle phase transition and DNA repair pathways. It is suggested that the expression of genes related to the immune activation pathway is active after immunizing mice with Ddx21MT.

## 4 Discussion

Tumor-reactive T cells have long been regarded as a promising therapeutic approach for regulating tumor growth and eradicating tumors (25, 26). Cytotoxic T cells (CTLs) have the capability to identify mutated proteins presented on the surface of tumor cells as short peptides bound to major histocompatibility complex (MHC) molecules (27–29). These short peptides may come from two categories of tumor antigens, namely tumor-associated antigens (TAAs) and tumor-specific antigens (TSAs). TAAs are antigens that are overexpressed in tumor cells but may also be expressed at low levels in normal tissues. Therefore, targeting TAAs for treatment may lead to adverse effects on normal tissues (30, 31). On the other hand, TSAs are distinct protein sequences encoded by mutated genes in tumor cells that are not expressed in normal tissues. T cells have not undergone negative selection against these sequences, making them less tolerant compared to TAAs that are shared by both tumors and normal tissue (32). Because of this, neoantigens are crucial targets for immunotherapy, and the exploration of tumor vaccines based on neoantigens holds significant promise. However, their efficacy in clinical trials remains constrained. Many clinical trials of tumor vaccines have utilized target antigens or vaccine designs with insufficient immunogenicity to elicit immune responses (33). Therefore, the identification of priority targets for new epitopes is a crucial aspect in the development of neoantigen-based vaccines.

Here we investigate the neoantigens in a prophylactic lung cancer vaccine. We utilized two distinct cell lines, KPL 160302S and KPL 160424S, originating from different stages of lung cancer, as vaccines to immunize mice with spontaneous lung cancer. This will make up for the lack of research on preventive vaccines for lung cancer. Although both the KPL 160302S and KPL 160424S cell lines harbor the same driver mutations as the *Kras^LSL-G12D/+^*/*p53^LSL-R172H/+^* mice, they exhibit significant disparities in their immune responses. The vaccine based on KPL 160302S demonstrates a significant extension in the survival period of mice with lung cancer, whereas the vaccine produced by KPL 160424S does not yield the same effect. Compared to the control group of mice immunized with PBS, the median survival time of mice immunized with KPL 160302S vaccine was increased by 21% to 283 days. However, there was no significant extension in the median survival time of mice in the KPL 160424S vaccine group (236 days) (19). The immunohistochemical results of lung tissues from immunized mice revealed distinct pulmonary immune responses elicited by vaccination of the two different lung cancer cell lines. In the KPL 160302S group of mice, there was a persistent increase in the infiltration of CD8^+^ T cells into lung tissue for 1-3 weeks after booster immunization, as well as an elevated influx of CD4^+^ T cells into lungs within 1-2 weeks after booster immunization (19). The flow cytometry results indicate that the KPL 160302S vaccine significantly enhances TCM within CD8^+^ T and CD4^+^ T cell populations in spleens. These findings suggest that the KPL 160302S vaccine is more effective in eliciting anti-tumor immune response.

Identifying the key factors that affect the efficacy of tumor vaccines is of great significance for making efficient and stable tumor vaccines. The two cell lines, from the same mouse strain and carrying identical tumor-driving mutations, are used to produce the vaccine using a standardized method. This approach helps reduce variations in immune responses caused by differences in species, tumor mutations, or vaccine manufacturing methods. Moreover, the expression levels of MHC-I in KPL 160302S and KPL 160424S showed no significant difference, and they were confirmed to be free from mycoplasma contamination (data not shown), thus ruling out any potential impact on immune response. In summary, we believe that the variations in effectiveness between KPL 160302S and KPL 160424S as prophylactic vaccines may be attributed to additional mutations beyond the consistent driver mutations. Since these two cell lines were derived from lung cancer tissues obtained from *Kras^LSL-G12D/+^*/*p53^LSL-R172H/+^* mice at 8 weeks or 16 weeks after Ad-cre administration, respectively, they have undergone distinct periods of immune selection *in vivo*, leading to diverse mutation profiles. These various mutations have resulted in the generation of different neoantigens, leading to variations in immunogenicity between the two cell lines. We postulate that certain neoantigens originating from early-stage lung cancer tumor cell lines may hold significant promise for prophylactic vaccines.

Among numerous mutations, only a limited number of neoantigens are capable of eliciting anti-tumor immune responses (34–37). Therefore, we employed bioinformatics methods to forecast the key neoantigen epitopes in the prophylactic vaccine. According to the reported methods for predicting novel tumor antigens (38), we conducted a screening of potential neoantigens for KPL 160302S and KPL 160424S. Subsequently, we identified 12 promising neoantigens from KPL 160302S to assess their ability to elicit anti-tumor immune responses. An *in vitro* IFNγ release assay is frequently employed to assess the immunogenicity of potential neoantigens (25, 34, 38, 39). After immunizing *Kras^LSL-G12D/+^*/*p53^LSL-R172H/+^* mice with KPL 160302S/KPL 160424S/PBS, the splenocytes were co-incubation *in vitro* with 12 candidate neoantigen peptides and their corresponding wild-type peptides for 3 days. In comparison with the corresponding wild-type peptides, Ddx21MT and Zfp760MT significantly enhanced IFNγ secretion in splenocytes of all three mice in the KPL 160302S vaccinated group, as well as induced a significant increase in IFNγ secretion in one of three mice in the KPL 160424S vaccinated group. Additionally, Ddx21MT notably increased IFNγ secretion in one out of three mice in the PBS group. It can be inferred that after immunization with KPL 160302S, mice produced memory T cells in response to the immunogenic epitope contained in KPL 160302S. When these memory T cells were re-stimulated by the neoantigens in KPL 160302S *in vitro*, they would elicit a robust immune response. In addition, since the vaccinia virus is the vaccine adjuvant used to booster immunization, we also used B8R, an antigenic peptide of the vaccinia virus, to detect the immune system’s memory response to it in the IFNγ stimulation experiment *in vitro*. Three mice in the KPL 160302S vaccine group and three mice in the PBS control group had no immune response to B8R, while two of the three mice in the KPL 160424S vaccine group were stimulated by B8R to significantly higher IFNγ secretion. It was speculated that mice immunized with KPL 160302S had reduced memory of immune adjuvants and more tumor-specific T cells than mice immunized with KPL 160424S, thus facilitating tumor clearance.

In order to further verify the effect of Ddx21MT/Zfp760MT on the anti-tumor immune response of mice, we used Ddx21MT/Zfp760MT with adjuvant Poly-IC to immunize wild-type 129 mice. After 1 week of booster immunization, results of flow cytometry showed that the number of TCM in spleens, draining lymph nodes and lungs of Ddx21MT immunized mice increased significantly. TCM has a strong capacity for *in vivo* expansion, persistence, and anti-tumor properties (40). Meanwhile, it’s confirmed that splenocytes of Ddx21MT immunized mice secreted high IFNγ stimulated by lung cancer cell lines and Ddx21MT peptide. We speculated that Ddx21MT neoantigen is beneficial to long-term anti-tumor memory. To explore the molecular mechanism of Ddx21MT neoantigen stimulation of the immune system, CD8^+^ T and CD4^+^ T cells in the spleens after immunization were sequenced. The results of transcriptome sequencing indicated that the expression of *S1pr1* and *Mpzl1* in CD8^+^ T cells and CD4^+^ T cells is up-regulated in the Ddx21MT group compared to the Ddx21WT group. In addition, the expression of *Mmp11, Sult1a1*, and *Gdpd1* in CD8^+^ T cells and CD4^+^ T cells in the Ddx21MT group is also significantly higher than that in the PBS group. Some of these genes have been reported to be related to immune functional regulation.

Sphingosine-1-phosphate *(S1P)* receptors are a group of G-protein-coupled receptors that are currently divided into five subtypes, including *S1pr1*. *S1pr1* is highly expressed in T and B lymphocytes. It has been reported that high expression of *S1pr1* is associated with the migration of T cells from the thymus, and that *S1pr1* expression is down-regulated during the activation of T cells in peripheral immune organs. *S1pr1* plays an important role in the migration of T cells and B cells in the thymus and peripheral lymphoid organs (41). This is consistent with our findings that up-regulation of this gene in the Ddx21MT group can promote the increase of TCM in spleens, draining lymph nodes and lungs.

*Mpzl1* is a cell adhesion molecule known to play a role in the chemotaxis of leukocytes (42), and its function of regulating cell migration depends on both its cytoplasmic immunoreceptor tyrosine inhibitory motif (ITIM) and its interaction with the tyrosine protein phosphatase, src homology phosphatase-2 (SHP-2) (43). Our findings indicated an up-regulation of *Mpzl1* expression in CD4^+^ T cells and CD8^+^ T cells within the spleens of Ddx21MT-immunized mice, suggesting a potential role for high *Mpzl1* expression in T cell migration to tumor sites, thereby contributing to anti-tumor immunity.

In addition, an enrichment analysis of differential gene expression was conducted on CD8^+^ T cells and CD4^+^ T cells from the Ddx21MT immunized group and the control group. The results revealed that the differentially expressed genes were primarily associated with lymphocyte differentiation, lymphocyte migration, response to IFNγ, T cell mediated immunity, regulation of immune effector process, antigen processing and presentation, as well as other immune activation-related pathways. This further confirms that immunization of mice with the novel antigen Ddx21MT significantly activates the anti-tumor immune response.

In conclusion, we have identified a tumor neoantigen, the Ddx21MT (SNFPIFCDL), originating from early-stage lung cancer development in *Kras^LSL-G12D/+^*/*p53^LSL-R172H/+^* mice delivered with Ad-Cre. The Ddx21MT significantly increases CD8^+^ and CD4^+^ central memory T cells and enhances IFNγ secretion by splenocytes *in vitro*. However, the MHC of humans and mice is different, so the direct application of this neoantigen to clinical patients has certain problems. In fact, we want to emphasize the idea that early tumors undergo short immunoscreening time *in vivo*, and may have more immunogenic neoantigens for the production of lung cancer vaccines. The neoantigens obtained from patients with early stage tumors by early diagnosis, such as circulating tumor DNA (ctDNA), may have a stronger activation effect on the immune system than the neoantigens obtained from the patients with advanced tumors. The Ddx21MT has the potential to be developed into a prophylactic lung cancer antigen vaccine and could be used with other biological therapies for lung cancer prevention and treatment.

## Data Availability

The datasets presented in this study can be found in online repositories. The names of the repository/repositories and accession number(s) can be found in the article/**Supplementary Material**.
