# Supplementary material for: Ddx21 mutant peptide is an effective neoantigen in prophylactic lung cancer vaccines and activates long-term anti-tumor immunity
Source: Front Immunol. 2025 Feb 6;16:1500417. doi: 10.3389/fimmu.2025.1500417 (PMC11839773; doi:10.3389/fimmu.2025.1500417)
Supplement: Supplementary file 1 [file DataSheet1.docx]

**Supplementary Figures**


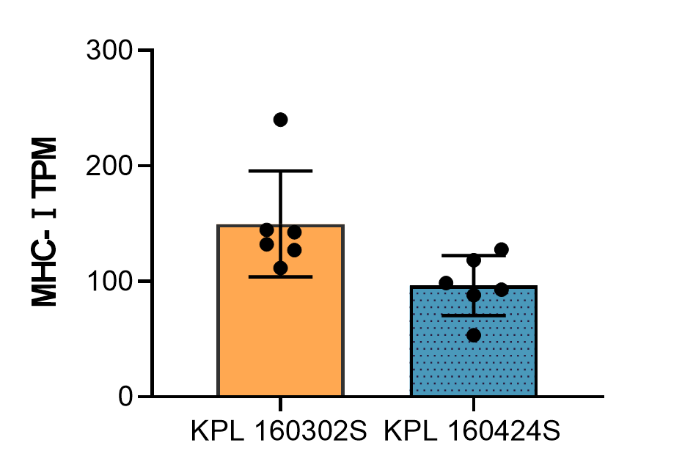


**Figure S1** Expression of classical MHC-I molecules (H-2D, H-2K) in KPL 160302S and KPL 160424S.


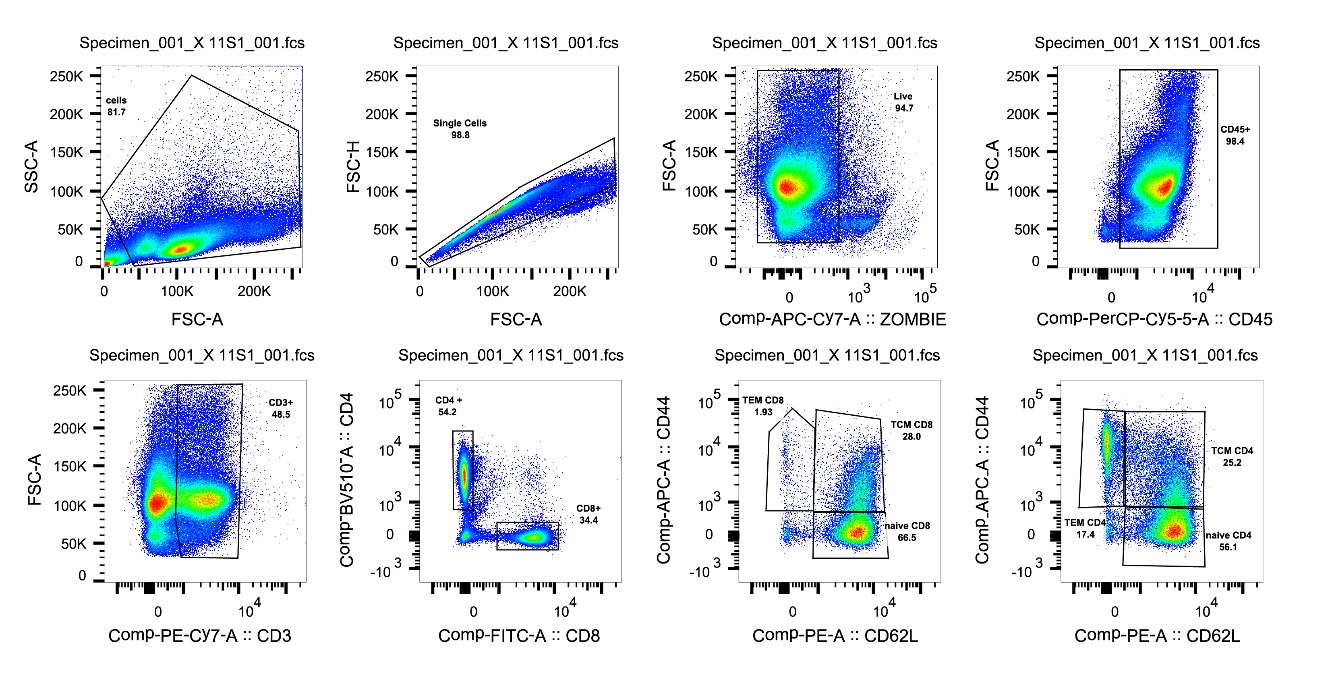


**Figure S2** TCM and TEM gating criteria are shown.


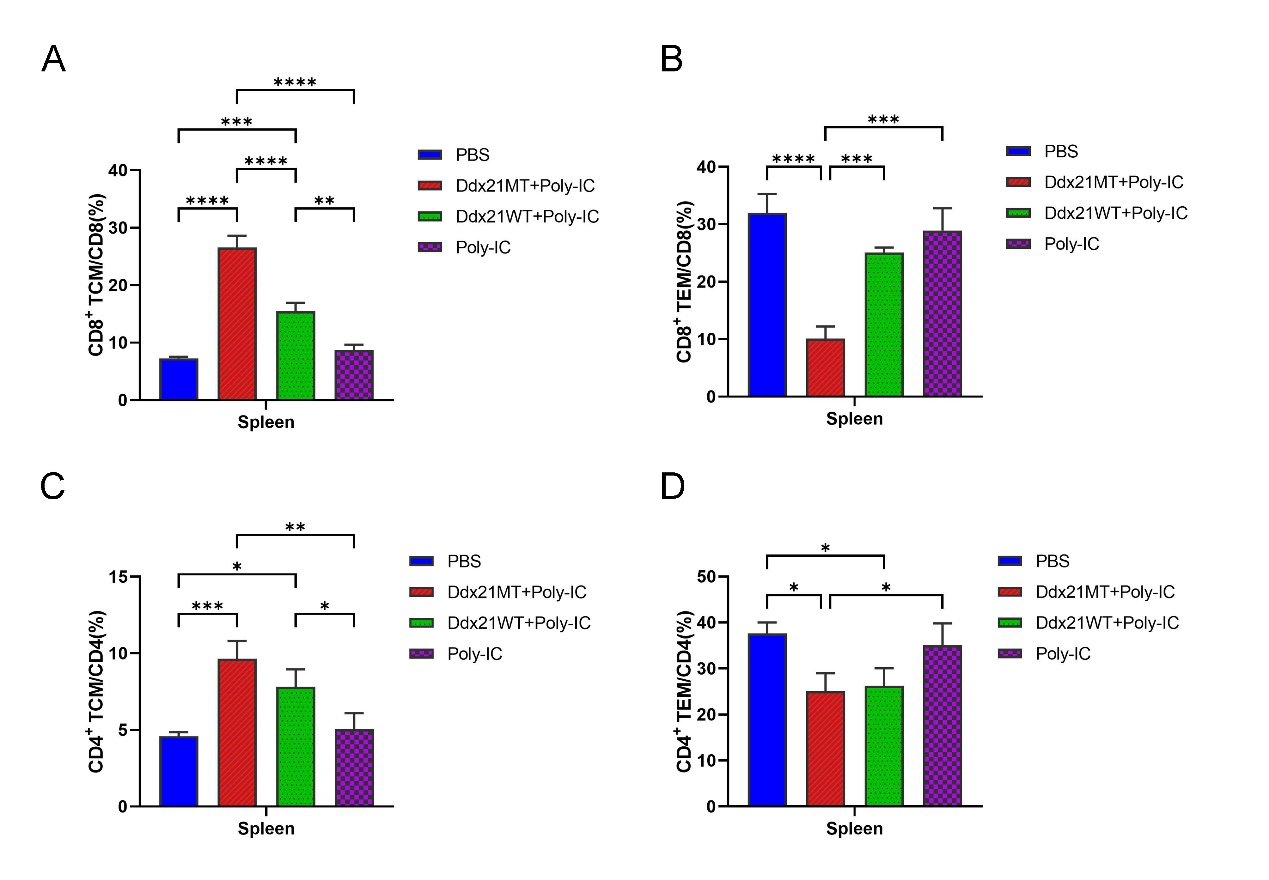


**Figure S3** Analysis of T cell subpopulations in spleens of B6 mice one week after immunization with two neoantigens and corresponding wild-type peptides. (A) Proportion of CD8^+^ TCM cells among CD8^+^ cell population in spleens. (B) Proportion of CD8^+^ TEM cells among CD8^+^ cell population in spleens. (C) Proportion of CD4^+^ TCM cells among CD4^+^ cell population in spleens. (D) Proportion of CD4^+^ TEM cells among CD4^+^ cell population in spleens. (One-way ANOVA and multiple comparisons between groups, n=3, ****p < 0.0001, *** p < 0.001, **p < 0.01, *p < 0.05).
